# The Athlete Gut Microbiome: A Narrative Review of Multi-Omics Insights and Next-Generation Probiotic Strategies

**DOI:** 10.3390/nu17203260

**Published:** 2025-10-17

**Authors:** Zhiwei Li, Youqiang Li, Yufei Wang, Jinjin Chen, Yilan Liu

**Affiliations:** 1 Department of Physical Education, Nanjing Railway Vocational and Technical College, No. 65 Zhenzhu South Road, Pukou District, Nanjing 210031, China; 2121151101@sus.edu.cn; 2 School of Physical Education, Shanghai University of Sport, 399 Changhai Road, Yangpu District, Shanghai 200438, China; youmecity@163.com; 3 Department of Physical Education, Donghua University, No. 1882 West Yan’an Road, Shanghai 200051, China; wangyufei@dhu.edu.cn; 4 Department of Chemical Engineering, University of Waterloo, 200 University Avenue West, Waterloo, ON N2L 3G1, Canada

**Keywords:** multi-omics, gut microbiome, probiotics, athletic performance

## Abstract

The gut microbiome plays a pivotal role in athletic health and performance by influencing metabolism, immunity, gastrointestinal integrity, and recovery. Advances in high-throughput sequencing and integrative multi-omics approaches have provided unprecedented insights into how microbial communities adapt to the physiological demands of training and competition. Key microbial taxa, including short-chain fatty acid producers, lactate utilizers, and carbohydrate fermenters, have been linked to enhanced endurance, reduced inflammation, and improved recovery, opening exciting opportunities for targeted probiotic interventions. While conventional probiotics remain valuable, next-generation engineered strains hold particular promise, supported by recent regulatory milestones such as FDA approvals of engineered probiotics and gene-edited livestock. In this narrative review, we synthesize recent multi-omics research to examine mechanistic links between the athlete gut microbiome and performance, evaluate current and emerging probiotic strategies, and highlight challenges related to personalization, standardization, and regulation. With longitudinal, performance-oriented trials and harmonized frameworks, microbiome-targeted interventions are poised to move beyond exploratory research toward evidence-based, precision tools that optimize athletic performance and recovery.

## 1. Introduction

Over the past few decades, the gut microbiome has emerged as a critical component of human health, with influence across immunity, energy metabolism, neurocognitive signaling, and tissue repair. Conversely, the makeup and function of gut microbial communities are shaped by diet, lifestyle, antibiotic and other medication use, hygiene, and the host’s genetic and immune background [1,2]. Athletes, expose this microbial community to exceptional stresses, such as rapid dietary shifts, high training loads, long-haul travel, abrupt weight cycling, extreme heat or altitude, and routine use of supplements or non-steroidal anti-inflammatory drugs. These pressures continually remodel the gut ecosystem in ways that can either fortify barrier integrity and dampen exercise-induced inflammation or, when dysregulated, impair recovery and diminish performance [3]. As such, research into the athletic gut microbiome is increasingly recognized as essential for understanding host–microbe interactions under physiological stress and for identifying microbiome-based strategies to enhance athletic health, recovery, and performance.

With the rapid advancement of biological technologies and the steadily decreasing cost of testing, research into the athletic gut microbiome has accelerated significantly. High-throughput DNA/RNA sequencing, along with protein, lipid, and metabolite profiling, has become more accessible, enabling comprehensive multi-omics approaches [4]. Metagenomics reveal the genetic potential of gut microbes, while metatranscriptomics and metaproteomics uncover which genes and proteins are actively expressed during training or competition. Metabolomics further captures the dynamic exchange of molecules, such as short-chain fatty acids (SCFAs) and neurotransmitters, between microbes and the host that influence physiological function. By integrating data from multiple sources, including saliva, blood, and stool, researchers can begin to correlate microbial activity with key performance traits like endurance, strength, recovery, and immune resilience [5]. The application of machine learning and artificial intelligence to these complex datasets is further accelerating discovery, offering new insights into how the microbiome can be modulated to support athletic performance [6,7].

**Figure 1:**
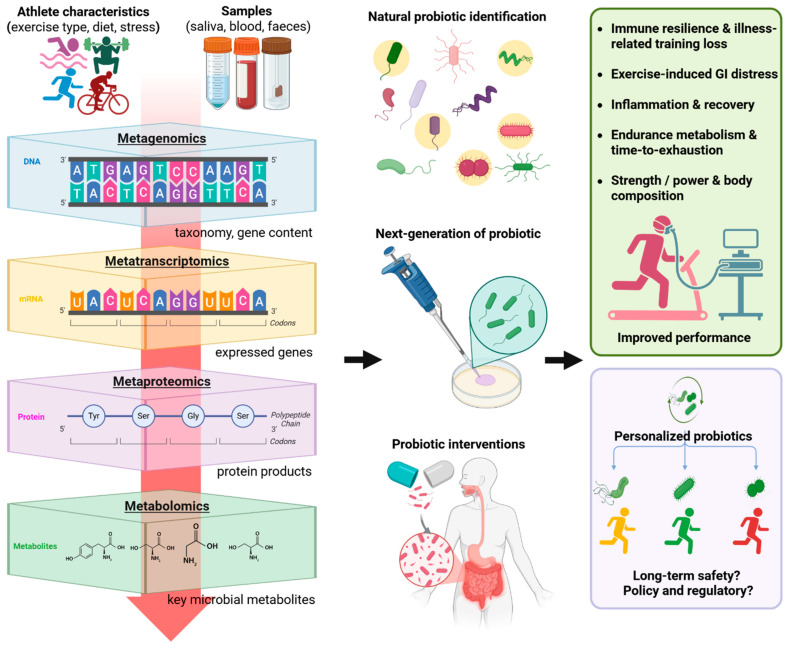
Overview of multi-omics framework for decoding the athlete gut microbiome and guiding probiotic interventions.

As shown in Figure 1, the multi-omics approach transforms complex biological data into actionable insights, enabling the identification of microbial signatures linked to improved athletic performance. This knowledge paves the way for the development of next-generation probiotics [8,9], including genetically engineered strains tailored to enhance specific physiological outcomes such as endurance, recovery, or immune resilience. These naturally selected or engineered probiotics can be delivered through targeted interventions aimed at modulating the gut microbiome to support athletic demands. As analytical tools and AI-based integration of omics data improve, the potential for truly personalized probiotic strategies becomes increasingly attainable, allowing interventions to be matched to an athlete’s unique microbiome, training profile, and health status. However, these advances also raise important questions around long-term safety, regulatory oversight, and fairness in competitive sports.

This review synthesizes recent multi-omics research to examine the dynamic interplay between the athlete’s gut microbiome and physical performance. We first present an overview of omics-based studies that profile microbial composition and function in athletes. Next, we explore mechanistic insights into how gut microbes influence exercise-related outcomes such as endurance, recovery, and inflammation. We then evaluate current and emerging probiotic interventions designed to enhance athletic performance, highlighting their potential applications and limitations. Finally, we discuss key challenges and research gaps, including issues of safety, efficacy, personalization, and regulatory oversight. Our aim is to provide researchers, sports scientists, and nutrition professionals with a clear and integrated framework for translating microbiome science into effective, evidence-based strategies to optimize athletic health and performance.

## 2. Omics Studies of the Athlete Gut Microbiome

The gut microbiome’s role in athletic performance has gained significant attention, driven by its influence on energy metabolism, immune function, and recovery processes. Recent advances in sequencing and bioinformatics, coupled with reduced costs, have made microbiome profiling more accessible to researchers and clinicians. Multi-omics approaches, including metagenomics, metatranscriptomics, metaproteomics, and metabolomics, provide an integrative framework to dissect how gut microbial ecosystems adapt to the physiological demands of athletes during training, dietary transitions, and environmental stress [10]. Metagenomic analyses reveal microbial species, and genes involved in key metabolic and signaling pathways. Transcriptomic data indicates that these genes are actively expressed in response to exercise and diet, while proteomic and metabolomic analyses confirm the translation of these signals into functional proteins and metabolites that modulate host physiology. Together, these omics layers provide a coherent systems-level view of how microbial functions translate into host performance benefits. Table 1 summarizes the meta-omics techniques, their main focus and connections, sample types, instrumentation, cost levels, and associated performance or health endpoints.

### 2.1. Metagenomics: Taxonomic and Functional Gene Profiles

Metagenomics enables high-resolution analysis of microbial composition and function by sequencing total community DNA [11]. Common approaches include 16S rRNA gene sequencing for cost-effective diversity profiling and shotgun metagenomics for detailed species- and gene-level insights. With sequencing costs now as low as US $30 per sample, large-scale studies are feasible. However, contamination and variability in athlete diets and environments require standardized protocols for sample collection, storage, and sequencing to ensure data reliability and cross-study comparability [12,13]. These guidelines are critical for reproducibility and cross-study comparability in gut microbiome research.

Metagenomic studies have identified microbial signatures associated with athletic performance, such as specific taxa like *Veillonella* that correlate with physiological metrics like VO_2_max, lactate threshold, and recovery efficiency [14]. A large-scale meta-analysis of 418 metagenomic datasets further revealed that athletes exhibit increased α- and β-diversity, along with enrichment of SCFA-producing genera such as *Faecalibacterium*, *Eubacterium*, *Blautia*, and *Ruminococcus* [15]. Genera such as *Bacteroides* and *Prevotella* play pivotal roles in host metabolic processes, including carbohydrate fermentation and SCFA production, which are critical for supporting energy demands, muscle repair, and endurance in elite athletes [16]. These findings provide a static snapshot of microbial potential, setting the stage for deeper functional insights from other omics layers.

### 2.2. Metatranscriptomics and Metaproteomics: Active Pathways and Expressed Proteins

While metagenomics outlines the genetic blueprint of the gut microbiome, meta-transcriptomics and metaproteomics reveal which genes and proteins are actively ex-pressed under the specific conditions of athletic training and competition [16,17,18]. However, their use in sports science remains limited due to high technical and financial barriers. Metatranscriptomics requires RNA stabilization, enrichment, and deep sequencing to overcome RNA scarcity and host contamination [19], while metaproteomics demands complex extraction and LC–MS/MS analysis to detect low-abundance microbial proteins [18]. Both methods provide valuable functional insights but remain costly and challenging, typically exceeding US $300 per sample.

Despite these hurdles, pioneering studies demonstrate the value of metatranscriptomics and metaproteomics in uncovering training- and competition-driven microbial shifts in athletes. Using metatranscriptomics combined with metabolomics, one study demonstrated that *Veillonella* blooms post-marathon upregulate lactate-to-propionate conversion genes that enhanced endurance in mouse models [14]. Transcripts of archaeal methane production were detected in professional cyclists, suggesting a role in energy metabolism [20]. More recently, metaproteomics revealed an SCFA-rich protein signature in athletes that promoted muscle glycogen resynthesis in germ-free mice, highlighting direct links between microbial activity and recovery [21]. As sample stabilization kits and collaborative consortia lower costs and improve access, metatranscriptomics and metaproteomics are poised to shift the field from static gene catalogs to dynamic, activity-based insights, revealing how microbial functions adapt to the physiological demands of exercise.

### 2.3. Metabolomics: SCFAs, Amino Acid Metabolites, and Others

Metabolomics quantifies small molecules, such as amino acids, sugars, lipids, nucleotides, and organic acids, produced by the gut microbiome and exchanged with the host, offering a direct window into the metabolic outcomes of microbial activity [22]. Using LC-MS/MS or Gas chromatography–mass spectrometry (GC-MS), metabolomics is relatively cost-effective (≤US $100 per sample) and widely accessible, making it a popular complement to metagenomics. Key metabolites, such as SCFAs (e.g., butyrate, propionate), branched-chain amino acids (BCAAs), polyamines, bile acids, and the kynurenine:tryptophan ratio, reflect microbial contributions to energy metabolism, inflammation, and recovery [22]. In addition to targeted analyses, recent studies have used untargeted metabolomics (e.g., GC×GC–MS, RP–MS, NMR) to detect a wider range of metabolites. For example, untargeted RP–MS metabolomics was applied to 24-h ultramarathon runners and identified broad biochemical shifts in lipid oxidation, amino acid, and vitamin metabolism, uncovering novel metabolic markers correlated with performance [23]. Together, these molecules serve as biomarkers for training load, fatigue, adaptation, and overtraining risk, providing actionable insights for optimizing athletic performance.

Integrated multi-omics approaches link microbial genes, their expression, and metabolic outputs to reveal host–microbe interactions during exercise. Combined metagenomics, metatranscriptomics, and metabolomics can trace how *Veillonella* converts lactate into propionate, which reduces inflammation and boosts endurance [14]. Similarly, metagenomics and metabolomics analyses of rugby players revealed that the gut microbiota drive SCFAs production, with SCFAs linked to enhanced performance and immune modulation [24]. These integrated datasets enable researchers to map functional pathways, identify performance-enhancing microbes, and design targeted probiotics. Metagenomics remains the most widely used due to its affordability and robustness, while metatranscriptomics and metaproteomics are emerging as costs decline and protocols improve.

## 3. Mechanistic Connections Between Gut Microbiota and Athletic Performance

A comprehensive literature search was conducted in Google Scholar using the keywords “Omics,” “athlete,” “gut microbiota,” and “performance,” covering publications from 2014 to the present. The search yielded about 3250 papers, from which we screened and selected around 20 original research articles of high relevance and citation impact. Based on these studies and related reviews, we identified five functional domains through which the gut microbiota supports athletic performance: immune resilience, inflammation and recovery, gastrointestinal (GI) integrity, endurance metabolism, and strength, power and body composition. The subsections that follow synthesize findings across these domains and highlight how distinct microbial functions contribute to physiological adaptations that underpin athletic performance.

### 3.1. Immune Resilience and Illness-Related Training Loss

The gut microbiota plays a critical role in bolstering immune resilience, reducing the incidence and duration of illnesses that can disrupt athletic training and competition performance. Upper respiratory tract infections (URTIs) are a common setback for endurance athletes, often triggered by the immunosuppressive effects of intense exercise [25]. A prospective cohort study in elite UK athletes with 16S rRNA sequencing showed that athletes susceptible to RTI had airway dysbiosis, reduced memory T-regulatory cells, and sphingolipid pathway dysregulation, directly tying microbial and immune profiles to lower immune resilience [26]. Multi-strain probiotic supplementation, particularly with *Lactobacillus* and *Bifidobacterium* species, has been shown to mitigate these risks. Studies demonstrate that such interventions normalize tryptophan-kynurenine metabolism, a pathway linked to immune regulation, and increase salivary immunoglobulin A (IgA) levels, a key marker of mucosal immunity [27,28]. These findings align with reviews highlighting that beneficial microbiota configurations may buffer athletes against infection and illness-related training loss [29].

### 3.2. Inflammation and Recovery

Post-exercise inflammation and delayed-onset muscle soreness (DOMS) can hinder recovery, impacting subsequent training and performance. SCFAs, particularly butyrate, play a pivotal role in mitigating these effects [30]. Produced by fiber-fermenting taxa like *Faecalibacterium*, *Roseburia*, and *Akkermansia*, butyrate fuels colonocytes, strengthens tight junctions, and promotes regulatory T-cell expansion, reducing gut permeability and systemic cytokine surges. A landmark study in rugby players demonstrated that athletes had a higher diversity of gut microorganisms that correlated with lower systemic inflammation, and enriched *Akkermansia* abundance inversely correlated with metabolic disorders [31]. Building on the same rugby cohort, metagenomics and metabolomics revealed that athletes had greater microbial diversity and enriched SCFA-producing pathways, with elevated fecal SCFAs linked to dietary intake, muscle activity, and reduced systemic inflammation [24]. Extending these findings, a cohort of 543 multi-sport athletes revealed that sport type shaped discrete microbial subgroups, with a *Prevotella*-centric enterotype tracking systemic inflammation markers and sex further modifying these relationships [32]. Together, these studies highlight that the anti-inflammatory and barrier-strengthening functions of the microbiota contribute to reduced DOMS and accelerated recovery, underscoring its role in optimizing performance.

### 3.3. GI Integrity

Exercise-induced GI distress, including bloating, cramping, and nausea, is a common challenge for endurance athletes, particularly during prolonged events like marathons. These symptoms are often linked to compromised gut barrier integrity caused by heat stress, dehydration, and repetitive mechanical impact [33]. Evidence from ultra-endurance rowing shows that microbial α-diversity increased, with greater abundance of butyrate-producing taxa such as *Roseburia* and *Subdoligranulum*, which strengthen gut barrier integrity and correlate with reduced GI distress [34]. By contrast, elite skiers displayed α-diversity comparable to controls but reduced levels of mucin-degrading taxa (*Akkermansia*, *Bacteroides*, *Bifidobacterium*, *Ruminococcus*), a pattern suggestive of heightened GI stress [35]. Probiotic interventions offer a promising strategy to mitigate these issues. In a randomized, double-blind, placebo-controlled trial, 24 recreational marathon runners who received a four-week supplementation with the “Lab4” consortium (*Lactobacillus* and *Bifidobacterium*) reported significantly fewer moderate-to-severe GI symptoms during the race. Supplemented athletes experienced less bloating and cramping and maintained a steadier pace in the final third of the marathon compared to placebo [36]. The benefits are thought to arise from improved gut barrier function, as probiotics reinforce tight junctions and reduce endotoxin translocation. Collectively, these findings highlight the potential of microbiota-targeted interventions to reduce GI discomfort and support endurance performance under physiological stress.

### 3.4. Endurance Metabolism and Time-to-Exhaust

The gut microbiota enhances endurance performance by modulating energy metabolism, particularly through the recycling of exercise-derived substrates such as lactate. A landmark study demonstrated that *Veillonella atypica* blooms in marathon runners post-race, converting luminal lactate into propionate, a metabolite that boosts energy availability [14]. Mouse models gavaged with *Veillonella* or rectal propionate exhibited extended treadmill run times, providing causal evidence of lactate shuttling as a performance-enhancing mechanism. Similarly, transplantation of gut microbiota from elite professional cyclists into mice altered the hosts’ metabolic profiles and endurance, signaling that athlete-specific microbiomes can modulate energy metabolism to enhance performance [21]. In competitive cyclists, *Prevotella*-dominant microbiomes were enriched for branched-chain amino acid and carbohydrate metabolism pathways, while professional cyclists exhibited elevated transcriptional activity of *Methanobrevibacter smithii*, associated with methane, carbohydrate, and energy metabolism, collectively supporting prolonged time-to-exhaustion [20]. Together, these findings indicate that endurance-associated microbes enhance host substrate utilization and energy efficiency, thereby extending athletic time-to-exhaustion.

### 3.5. Strength, Power and Body Composition

Intestinal bacteria can affect strength, power, and body composition in athletes by producing a variety of biologically active metabolites, such as the best-known SCFAs, which correlate with greater lean mass, grip strength, and muscle fiber [37]. A study compared female athletes, anorexia nervosa inpatients, and women across BMI groups, and found that athletes displayed the highest gut microbial diversity, whereas obese and anorexic participants showed markedly reduced diversity. Greater microbial diversity in athletes was associated with healthier body composition and metabolic profiles, suggesting that microbial richness supports favorable muscle and body composition outcomes [38]. Aya et al. (2025) reported that elite weightlifters exhibited gut microbiomes enriched in amino acid biosynthesis pathways, particularly for BCAAs, supporting muscle protein synthesis and strength adaptations [16]. In contrast, cyclists showed greater representation of carbohydrate- and fatty acid-related pathways, including SCFA production, consistent with enhanced oxidative metabolism and endurance capacity. Beyond these sport-specific distinctions, their integrative omics analysis emphasized that key genera such as *Bacteroides* and *Prevotella* are central to athlete metabolism, contributing to energy utilization, muscle recovery, and performance, with lipidomic profiling underscoring the microbiome’s role in lipid handling and cellular adaptation. Together, these findings highlight that the gut microbiome contributes not only to endurance but also to strength and body composition by tailoring metabolic outputs to the demands of different athletic disciplines.

## 4. Probiotic Interventions for Enhancing Athletic Performance

Multi-omics evidence (Table 2) shows the gut microbiome supports athletic performance by enhancing immune resilience, regulating inflammation and recovery, preserving gastrointestinal integrity, and optimizing metabolism, strength, and body composition. Core microbial guilds, such as SCFA producers, lactate utilizers, and carbohydrate fermenters, underscore opportunities for targeted probiotics. These insights drive the development of next-generation, sport-specific probiotic strategies, transforming microbiome science into practical tools for athletes.

### 4.1. Candidate Microbes

Table 3 summarizes representative candidate probiotics with functional links to athletic performance. General lactic acid bacteria (LAB), including *Lactobacillus*, *Bifidobacterium*, *Pediococcus*, *Leuconostoc*, *Streptococcus*, *Saccharomyces*, *Bacillus*, and *Enterococcus*, are widely used as probiotics for maintaining general human health [39]. Within this group, some strains also show specific evidence for enhancing athletic performance. For example, Lactobacillus and Bifidobacterium species remain foundational in sports settings, as they support gut-barrier integrity and immune modulation, reducing URTIs and GI discomfort that disrupt training [36,40,41]. Human randomized controlled trials (RCTs) with *Lactiplantibacillus plantarum* TWK10 and *Bifidobacterium longum* OLP-01 further demonstrate direct performance benefits, including improved endurance, muscle strength, and favorable body composition [42,43].

Alongside these well-studied strains, newer candidates are gaining traction. *Akkermansia muciniphila*, first isolated in 2004, is enriched in elite athletes and widely recognized as a next-generation probiotic that supports gut barrier function, immunity, and metabolic efficiency [31,44]. While no RCTs have been conducted in athletes, a 12-week trial in elderly participants showed improved muscle strength, highlighting its promise for future sports applications [45]. *Escherichia coli* Nissle 1917 (EcN) has been shown to help protect gut integrity and reduce oxidative stress during strenuous activity [46]. Outer membrane vesicles (OMVs) secreted by EcN could help reinforce intestinal epithelial barrier integrity by upregulating and relocating tight-junction proteins, counteracting pathogen-induced permeability and inflammation [47]. *Saccharomyces boulardii* may represent a promising candidate probiotic for enhancing athletic performance, given its ability to reinforce gut barrier integrity and its demonstrated capacity to increase VO_2_max and maximal running speed in animal studies [48,49].

SCFAs (e.g., acetate, butyrate, propionate) are increasingly recognized as key metabolites that enhance athletes’ immunity, support exercise recovery through anti-inflammatory activity, and provide additional energy substrates that contribute to improved exercise performance [50]. Several gut microbes are established SCFA producers that may contribute to athletic performance. *Veillonella atypica* plays a unique role by recycling exercise-derived lactate into propionate, a pathway directly linked to enhanced endurance capacity, while *Faecalibacterium prausnitzii* and *Roseburia* spp. are cornerstones of the gut microbiome due to their dominant roles in butyrate production and their status as markers of gut health and resilience [14,51,52]. *A. muciniphila*, though best known for mucin degradation, supports butyrate production indirectly by providing acetate and other substrates for cross-feeding butyrate-producing bacteria, reinforcing its importance in gut health and athletic potential [53].

**Table 3:** Functional classes and representative probiotic candidates with potential to enhance athletic performance.

Functional Type	Representative Strain(s)	Potential Athletic Benefit(s)	Ref.
Lactic acid bacteria (LAB)	*Lactobacillus*, *Bifidobacterium* (multi-strain)	Immune modulation, gut-barrier support, thus reducing URTIs and GI symptoms, improving training consistency.	[36]
	*Lactiplantibacillus plantarum* TWK10	Enhances energy metabolism, promotes muscle glycogen storage, Improved endurance, muscle strength, favorable body composition.	[42]
	*Bifidobacterium longum subsp. longum* OLP-01	Gut–muscle axis modulation, immune regulation, Improved running endurance and adaptation.	[43]
Gut-barrier strengthening strains	*Akkermansia muciniphila*	Mucin layer reinforcement, tight-junction signaling, better gut integrity, nutrient absorption, stability under endurance stress.	[31,44]
	*Escherichia coli* Nissle 1917	Tight-junction upregulation, OMV signaling, help reducing intestinal permeability (“leaky gut”) under physical stress.	[46]
	*Saccharomyces boulardii*	Reinforces tight-junction structure and epithelial integrity, it may benefit physical performance.	[48,49]
SCFA producers	*Veillonella atypica*	Converts lactate to propionate, enhanced endurance via lactate recycling and fueling.	[14]
	*Faecalibacterium prausnitzii*	A cornerstone of a healthy gut microbiome, supports gut barrier integrity and modulates inflammation through butyrate production, thereby may enhance recovery and performance resilience in athletes.	[51]
	*Roseburia* spp.	Enriched in active and athletic populations, contributes to butyrate production that fuels colonocytes, strengthens the gut barrier, and exerts anti-inflammatory effects, thereby supporting recovery and gastrointestinal stability during endurance training.	[52,54]
Carbohydrate & amino acid metabolism strains	*Prevotella copri*	Carbohydrate fermentation, amino acid metabolism, association with endurance and carb utilization, reduced muscular fatigue; context-dependent inflammation.	[20]
	*Methanobrevibacter smithii*	H_2_ scavenging, fermentation balance, potentially greater energy harvest; enriched in elite cyclists	[20]
	*Bacillus subtilis*; *Bacillus clausii*; *Bacillus coagulans*	Enhanced protein digestion, anti-DOMS activity, better protein utilization, reduced muscle damage, faster recovery revealed by RCTs	[55,56]
Bile acid transformation	*Clostridium scindens*, *Eubacterium* spp.	7α-dehydroxylation; FXR/TGR5 signaling, improved lipid and glucose metabolism, insulin sensitivity, mitochondrial activity, and gut repair	[57,58]

Notes: OMV, outer membrane vesicle; DOMS, delayed-onset muscle soreness; FXR, farnesoid X receptor; TGR5, Takeda G protein-coupled receptor 5.

Carbohydrate and amino acid metabolism strains also hold promise. *Prevotella copri* ferments complex carbohydrates and amino acids to match high training demands, though it may promote inflammation in some contexts [20,59]. An additional, more speculative candidate is the archaeon *M. smithii*, a hydrogen-consuming methanogen that facilitates more efficient fermentation of complex carbohydrates, thereby enhancing energy harvest by other gut microbes [20]. Spore-forming probiotics *Bacillus* spp. can improve protein digestion and reduce DOMS evidenced by RCTs, supporting recovery and protein utilization in athletes [56,60]. Beyond this, bile acid-transforming microbes such as Clostridium and Eubacterium regulate host lipid metabolism and mitochondrial efficiency through FXR/TGR5 signaling, offering an additional metabolic axis for performance optimization [57,58]. Despite promising mechanistic rationales, many probiotic interventions in athletic settings have failed to deliver clear performance gains. In a systematic review of 17 randomized controlled trials involving athletes and active individuals, the majority (11 studies) found no significant improvements in aerobic performance (9 trials) or muscular strength (2 trials) following probiotic supplementation, compared to placebo [61]. Similarly, in a 90-day trial of elite road cyclists, supplementation reduced some gastrointestinal symptoms and perceived exertion levels, but did not significantly alter VO_2_ max, time to fatigue, or inflammatory markers [62]. This gap highlights the limitations of natural strains and suggests that genetic engineering may be required to strengthen or combine beneficial functions, paving the way for next-generation engineered probiotics tailored to athletic performance.

### 4.2. Next-Generation Probiotics (NGPs)

Recent insights into microbial metabolic pathways that influence athletic performance, together with advances in omics technologies and synthetic biology, are opening the door to next-generation, engineered probiotics designed to deliver targeted benefits for athletes. Traditional probiotics, such as blends of Lactobacillus and Bifidobacterium, are valuable for supporting gut health and immunity but are not specifically tailored to the demands of sport. In contrast, NGPs aim for precision, leveraging omics-based insights to design microbes that address performance-related needs such as enhanced endurance, accelerated recovery, and improved metabolic efficiency.

For example, Bacillus subtilis has been engineered to function as a live oral probiotic capable of clearing excess circulating lactate, a proof-of-concept demonstrated in rodent models [63]. By enhancing lactate clearance, such an approach could help delay fatigue and accelerate recovery, offering direct benefits for athletic performance. Another study engineered EcN to produce butyrate, a key SCFA with broad health benefits [64]. In addition, our previous studies show that engineered EcN strains produce high concentrations of OMVs that preserve epithelial barrier function and attenuate inflammation [65,66]. For athletes, such engineered probiotics could serve as precision tools to support metabolic resilience, gut integrity, and recovery pathways.

Beyond individual engineered strains, a promising frontier is the development of personalized or sport-specific probiotics. Every athlete’s gut microbiome is unique, shaped by their sport, diet, and training regimen. Multi-omics profiling can map these microbial patterns, enabling the design of tailored probiotics that match performance demands. For instance, endurance athletes such as marathon runners with *Prevotella*-rich guts may benefit from probiotics that boost carbohydrate metabolism, while strength athletes with higher levels of *Alistipes* or *Blautia* could benefit from strains supporting BCAA metabolism [16]. Specific taxa may also serve different roles across sports, *Veillonella* to recycle lactate for endurance, or *Akkermansia* to support lean mass and metabolic efficiency in strength sports. This concept is reinforced by Li et al. [32], who documented distinct microbial signatures between different sports and noted sex-dependent microbial responses, underscoring the potential for highly personalized interventions.

Commercial momentum is already visible. ZBiotics (https://zbiotics.com; accessed on 1 October 2025) has brought the world’s first ‘proudly GMO’ probiotic to market, two engineered Bacillus subtilis probiotics: one designed to break down acetaldehyde from alcohol metabolism and another to convert sugar into fiber. Together they demonstrate growing consumer acceptance of genetically modified microbes, with more than 5 million bottles sold to date [67], showing growing public acceptance of engineered microbes. As highlighted in Nature, this trend reflects a broader shift in biotechnology, with the first engineered pigs expected to reach the market in 2026 [68]. While current engineered probiotics are not yet tailored to athletes, their success provides a blueprint for next-generation probiotics designed to enhance endurance, recovery, and overall athletic performance.

## 5. Limitations, Challenges, and Research Gaps

The study of the athlete gut microbiome has advanced through multi-omics approaches, yet several limitations restrict its reliability and broader application to sports performance. Sample collection practices vary across studies, with differences in storage conditions, preservation methods, and stool processing potentially introducing batch effects that reduce comparability [69]. Similarly, data processing pipelines are not yet standardized, as sequencing depth, reference databases, and bioinformatics workflows can influence taxonomic and functional outputs. These methodological differences have been noted as important sources of variability in microbiome research [70]. Although standardized protocols have been proposed [13], their adoption has been limited, making it difficult to compare across studies. Prediction represents another major barrier: incomplete metadata on training load, diet, and recovery obscure the context of microbial shifts, while current models struggle to integrate multi-omics data into reliable forecasts due to high costs, limited datasets, and computational challenges. Together, these gaps restrict the ability to move from correlation toward mechanistic understanding and performance-based prediction.

In applying microbiome science to athletics, three challenges stand out, yet each presents an opportunity for progress. Personalization remains key, as inter-individual variability in diet, sport type, sex, and genetics shapes microbial profiles and responses to interventions; while *Prevotella* or *Veillonella* may benefit some athletes more than others, this variability underscores the promise of precision strategies guided by multi-omics and AI. Verification is another hurdle, with many probiotic trials underpowered or lacking sport-specific endpoints such as VO_2_max or recovery time, but this gap is driving the design of larger, standardized, performance-oriented studies that will enable reproducible and actionable insights. Regulation also remains fragmented, particularly for genetically modified probiotics, yet recent FDA approvals of engineered microbes and gene-edited livestock demonstrate that oversight is evolving to support innovation while ensuring safety [67,68]. Far from being barriers, these challenges represent the next steps toward translation, with advances in personalization, rigorous validation, and harmonized regulation paving the way for microbiome-targeted interventions to become powerful, evidence-based tools for optimizing athletic performance and recovery.

## 6. Conclusions and Future Perspectives

Multi-omics research has revealed that the gut microbiome plays an active role in supporting athletic performance and recovery. By integrating metagenomics, metabolomics, metatranscriptomics and metaproteomics, studies consistently show that athletes harbor functional groups of microbes that enhance energy metabolism, reduce inflammation, strengthen gut integrity, and accelerate recovery. These include SCFA producers such as *Faecalibacterium* and *Roseburia*, lactate utilizers like *Veillonella*, and carbohydrate fermenters such as *Prevotella*. Gut-barrier stabilizers (*Akkermansia*, EcN, *Saccharomyces*) further protect against the gastrointestinal stress of endurance training. Collectively, these taxa demonstrate how microbial functions can be harnessed to improve training adaptation and resilience.

Translation into practice is already underway. Conventional strains (*Lactobacillus*, *Bifidobacterium*) improve immunity and training consistency, while targeted strains such as *L. plantarum* TWK10 and *B. longum* OLP-01 have demonstrated measurable benefits in endurance and muscle function. Looking ahead, engineered probiotics offer unprecedented potential. Recent FDA approvals of engineered microbes and gene-edited animals reflect a regulatory environment increasingly open to innovation, strengthening the path toward next-generation, performance-focused probiotics.

Remaining challenges include variability in individual responses, underpowered trials, and the need for standardized endpoints. However, rapid advances in multi-omics, AI-driven analytics, and synthetic biology are overcoming these barriers. With harmonized frameworks and longitudinal, performance-oriented studies, microbiome science is poised to deliver precision tools that enhance resilience, recovery, and performance in athletes.

## Figures and Tables

**Table 1:** Meta-omics techniques for athletic-performance studies.

Omics Layer	Main Focus/Connection with Other Omics	Athlete-Sample Matrix	Core Instrumentation	Cost Level *	Functions
Metagenomics	Microbial gene content and potential functions; Provides the blueprint confirmed by transcriptomics and proteomics	Fecal stool	Illumina NextSeq 2000/NovaSeq for shotgun; PacBio Vega or ONT PromethION for long-reads	Moderate ($$)	Reveals which microbes are present and the functional genes they encode (e.g., SCFA production, lactate utilization, vitamin biosynthesis), and how these features correlate with performance metrics such as VO_2_ max, lactate threshold, and recovery.
Metatranscriptomics	Microbial gene expression profiles; Links gene presence (genomics) to active function (proteomics and metabolomics)	Frozen or preservative-stabilized stool samples; serial sampling around endurance events	Illumina NextSeq 2000 stranded RNA-seq; optional PacBio Iso-Seq	High ($$$)	Shows which microbial genes are actively expressed under training stress, capturing dynamic pathways such as carbohydrate and SCFA metabolism that support glycogen replenishment and gut-barrier adaptation.
Metaproteomics	Protein abundance and enzyme activity; Bridges transcriptional activity and metabolite production	Stool protein extracts	LC-MS/MS on Orbitrap or Q-TOF	High ($$$)	Identifies microbial and host proteins produced, confirming functional shifts in enzymes, transporters, IgA, and stress proteins that reflect gut-barrier strain and host–microbe interactions.
Metabolomics	Small-molecule metabolites; Provides functional readout validating upstream omics layers	Stool, plasma/serum, dried-blood spots, urine; dense peri-exercise time-series	LC-MS/MS, RP-MS, GC-MS, GC×GC-MS, complemented by NMR	Low ($)	Measures a wide range of small metabolites (e.g., SCFAs, bile acids, BCAAs, acyl-carnitines) that provide real-time markers of training load, fatigue, adaptation, and overtraining risk.

* Cost levels: $ (Low): ≤≈US $100; $$ (Moderate): ≈US $30–300; $$$ (High): ≥≈US $300. Exact prices vary with read depth, MS mode, and volume discounts, but these tiers capture the typical relative expense hierarchy. LC-MS/MS: liquid chromatography–mass spectrometry; Q-TOF: quadrupole time-of-flight; RP-MS, reversed-phase chromatography–mass spectrometry; GC-MS: gas chromatography–mass spectrometry; NMR: nuclear magnetic resonance spectroscopy; SCFAs: short-chain fatty acids; BCAAs: branched-chain amino acids.

**Table 2:** Summary of Representative Studies on Athlete Gut Microbiota Using Omics Approaches.

No.	First Author (Year)	Omics Approaches	Cohort & Sport	Headline Gut-Microbiota Findings	Ref.
**Immune Resilience**					
1	Cuthbertson (2022)	16S rRNA sequencing Metabolomics	121 elite athletes	RTI-susceptible athletes displayed upper airway microbiome dysbiosis (reduced bacterial biomass and diversity), along with immunometabolic dysregulation in the sphingolipid pathway and reduced circulating memory T-regulatory cells, indicating a perturbed mucosal microbial–immune ecosystem linked to lowered immune resilience.	[26]
**Inflammation and recovery**					
2	Clarke (2014)	16S rRNA sequencing	40 rugby players, 46 controls	Early landmark showing athletes’ higher α-diversity that positively correlates with creatine kinase and lower systemic inflammation, and *Akkermansia* abundance inversely correlates with metabolic disorders.	[31]
3	Barton (2017)	16S rRNA sequencing Metabolomics	40 pro rugby players, 46 controls	Athletes showed greater gut microbial diversity and enriched SCFA-producing pathways (*Roseburia*), with higher fecal SCFAs linked to protein/fiber intake, creatine kinase, and reduced systemic inflammation, distinguishing them metabolically from controls.	[24]
4	Li (2023)	16S rRNA	543 multi-sport athletes	Sport-type shaped ten discrete microbial subgroups; a *Prevotella*-centric enterotype tracked systemic inflammation markers, and sex modified the exercise–microbiota relationship. Created athlete microbiota catalog; identified inflammation-risk signatures per sport.	[32]
**GI integrity**					
5	Keohane (2019)	16S rRNA sequencing	4 well-trained male athletes completing a trans-Atlantic, ultra-endurance rowing race	Ultra-endurance rowing increased gut α-diversity, abundance of certain species such as *Prevotella copri*, *Dorea longicatena*, and some butyrate producing species (*Roseburia* and *Subdoligranulum*) that linked to reduced inflammation and strengthened gut barrier function.	[34]
6	Hintikka (2022)	16S rRNA sequencing Metabolomics	27 elite skiers, 27 controls	Skiers exhibited a low-diversity microbiome characterized by depletion of mucin-degrading taxa (*Akkermansia*, *Bacteroides*, *Bifidobacterium*, and *Ruminococcus*), possibly reflecting GI stress during heavy training; *Butyricicoccus* was positively associated with higher HDL and HDL2 cholesterol and larger HDL particle size, suggesting its potential as a beneficial probiotic.	[35]
**Endurance metabolism**					
7	Petersen (2017)	Metagenomics Metatranscriptomics	33 competitive cyclists (18 professional, 15 amateur)	High-fitness athletes enriched for *Prevotella* species; *Prevotella*-dominant microbiomes associated with enhanced branched-chain amino acid and carbohydrate metabolism; exercise volume correlated with microbiota composition.	[20]
8	Scheiman (2019)	16S rRNA sequencing Metagenomics Metatranscriptomics Metabolomics	15 marathoners (pre/post), mouse validation	Post-race bloom of *Veillonella atypica*; strain isolated and shown to enhance treadmill endurance in mice via lactate-to-propionate metabolism	[14]
9	Martin (2025)	Metagenomics Metaproteomics Metabolomics	18 elite footballers/cyclists	Despite low overall diversity, athletes carried a protein-rich SCFA-producing guild; transplanting these communities into mice boosted muscle glycogen and insulin sensitivity, underscoring a causal link between microbial SCFA output and energy metabolism.	[21]
**Strength, power and body composition**					
10	Mörkl (2017)	16S rRNA sequencing	20 female athletes, 86 comparators	Athletes exhibited the highest gut α-diversity; diversity mirrored leanness and anti-inflammatory markers, highlighting exercise as a driver of beneficial gut ecology across body-composition strata.	[38]
11	Aya (2025)	Metagenomics Metabolomics Lipidomics	16 elite weightlifters, 13 cyclists	Weightlifters carried carnitine-utilizing guilds (enriched *Alistipes*, *Blautia*) and branched-chain-amino-acid gene modules, correlating with peak power; cyclists showed *Prevotella*/*Veillonella*-driven SCFA & triglyceride-hydrolysis pathways, tracking VO_2_max.	[16]
